# Microgravity and Human Body: Unraveling the Potential Role of Heat-Shock Proteins in Spaceflight and Future Space Missions

**DOI:** 10.3390/biology13110921

**Published:** 2024-11-13

**Authors:** Olga Maria Manna, Stefano Burgio, Domiziana Picone, Adelaide Carista, Alessandro Pitruzzella, Alberto Fucarino, Fabio Bucchieri

**Affiliations:** 1 Department of Biomedicine, Neurosciences and Advanced Diagnostics (BIND), Institute of Human Anatomy and Histology, University of Palermo, 90127 Palermo, Italy; 2 Euro-Mediterranean Institute of Science and Technology (IEMEST), 90146 Palermo, Italy; 3 Department of Medicine and Surgery, Kore University of Enna, 94100 Enna, Italy; 4 Department of Theoretical and Applied Sciences, eCampus University, 22060 Novedrate, Italy

**Keywords:** heat-shock proteins, microgravity, space medicine

## Abstract

In recent years, the increasing number of long-duration space missions has prompted the scientific community to undertake a more comprehensive examination of the impact of microgravity on the human body during spaceflight. This review aims to assess the current knowledge regarding the consequences of exposure to an extreme environment, like microgravity, on the human body, focusing on the role of heat-shock proteins (HSPs). Previous studies have demonstrated that long-term exposure to microgravity during spaceflight can cause various changes in the human body, such as muscle atrophy, changes in muscle fiber composition, cardiovascular function, bone density, and even immune system functions. It has been postulated that heat-shock proteins (HSPs) may play a role in mitigating the harmful effects of microgravity-induced stress. According to past studies, heat-shock proteins (HSPs) are upregulated under simulated microgravity conditions. This upregulation assists in the maintenance of the proper folding and function of other proteins during stressful conditions, thereby safeguarding the physiological systems of organisms from the detrimental effects of microgravity. HSPs could also be used as biomarkers to assess the level of cellular stress in tissues and cells exposed to microgravity. Therefore, modulation of HSPs by drugs and genetic or environmental techniques could prove to be a potential therapeutic strategy to reduce the negative physiological consequences of long-duration spaceflight in astronauts.

## Simple Summary

In the past few years, scientists have been studying the effects of microgravity on the human body during long-term space missions. They have found that being in space for a prolonged time can lead to muscle and bone complications, cardiovascular changes, and even a weaker immune system. One potential way to help the body cope with these changes involves heat-shock proteins. These proteins seem to help protect the body during stressful conditions, like those found in space. Studies have shown that HSPs are more active when cells are exposed to conditions similar to microgravity, and could, thus, be used to study stress reactions in space. In the future, researchers might be able to develop drugs or other techniques to change how these proteins work, helping astronauts stay healthier during long space missions.

## 1. Introduction

For many years, human beings have been motivated by the desire to explore and understand their role on Earth and in the universe, leading to extensive study of the space environment. Human space exploration has enabled knowledge transfer, improving Earth’s quality of life and health. Nevertheless, the unique conditions of space have required a comprehensive assessment of the potential risks to human health associated with both short-term and long-term exposure to this environment. The majority of space missions have taken place in low Earth orbit (LEO), located just a few hundred kilometers above the Earth’s surface. In LEO, objects remain in a perpetual state of free fall around the Earth, resulting in weightlessness for astronauts [1]. The International Space Station (ISS) is an example of a facility in LEO where astronauts reside and conduct scientific experiments investigating, among other subjects, the effects of weightlessness on the human body. The ISS provides a unique perspective for studying the space environment and its impact on the human body over extended periods [2]. Experiments carried out during spaceflight can be used for post-flight analysis on Earth, offering valuable insights into the long-term effects of space missions on human physiology. Extensive research and post-mission longitudinal studies on astronauts have yielded significant data on the environmental factors that most profoundly affect the human body after prolonged stays aboard the ISS. The main factors that can cause biological changes and serve as study targets on the ISS include radiation, confinement and isolation, extreme temperatures, circadian disruptions, and microgravity [3]. Astronauts experience apparent weightlessness in space due to microgravity, a condition where inertial forces balance the force of gravity. The International Space Station (ISS), orbiting Earth at an altitude of 330 to 410 km, is within a gravitational field that exerts 88.9% of the gravity felt on Earth’s surface. However, its constant velocity of 27,600 km/h results in free fall, leading to the sensation of weightlessness for astronauts. The physical and biological changes observed in space highlight the significant influence of gravity on human evolution on Earth [4].

The impacts of microgravity on the human body can be divided into two primary categories: effects that occur after short-term exposure, primarily consisting of space adaptation syndrome (SAS) and temporary nausea caused by an imbalance of the vestibular system; and effects that occur after long-term exposure [5].

In the absence of gravity, astronauts often experience confusion and disorientation due to conflicting signals from the central vestibular system, peripheral pressure receptors, and visual senses. This condition, known as space adaptation syndrome (SAS), is a common cause of space motion sickness (SMS) among astronauts, affecting approximately 70% of them during the initial days of orbital flight [6]. Symptoms include nausea, disorientation, and general malaise, which typically persist for 2–3 days after the transition from terrestrial gravity to microgravity [7]. The precise causes of SAS remain unclear; however, sensory conflict is believed to be a significant factor. This theory suggests that in microgravity, there is a discrepancy between the sensory information needed for movement and that given by the vestibular system, particularly when visual or body–position signals are conflicting. These discrepancies affect the otolithic channel and the interaction between visual and vestibular systems. A series of pharmacological countermeasures have been implemented to counteract SAS, such as the use of scopolamine and dextroamphetamine [8,9]. However, more recent research has reported that the most effective countermeasure is preliminary training on a virtual space station. The training device used for this research was NASA’s DOME (Device for Orientation and Motion Environments), which simulated sensory conflicts, like those occurring in space, allowing progressive habituation of the astronauts to these conditions [10].

Long-term exposure leads to structural and functional changes in the body, and research is crucial for safeguarding astronaut health on missions to the Moon and Mars. Due to the rapid advances in space engineering and technology, along with the increasing popularity of commercial spaceflight, it is crucial to investigate the effects of the space environment on the human body [11]. Nonetheless, our knowledge on the primary molecular pathways involved, and particularly the role of heat-shock proteins (HSPs), still remains limited.

This review provides an overview of the major challenges that long-duration spaceflight poses to the human body, with a specific focus on the role of heat-shock proteins (HSPs). It outlines the key risk factors associated with extended space travel, highlighting its impact on the human cardiovascular system, endothelial cells, musculoskeletal system, liver, and immune response. Drawing mainly on research from 2010 to 2023, the review also references historical spaceflight findings. It concludes by addressing the critical challenges and unresolved issues that must be tackled before extending human space exploration, with an emphasis on HSPs as potential biomarkers and therapeutic targets.

## 2. HSPs and Microgravity: Exploring the Role of HSPs in Cellular Adaptation to Spaceflight

Living organisms rely on the constant adaptation of cells, and maintaining homeostasis is essential for their survival and integrity. Heat-shock proteins (HSPs) are multimolecular complexes consistently expressed under physiological conditions and play a vital role in several cellular maintenance processes. These functions include properly folding newly synthesized polypeptides, the assembly of protein complexes, the degradation of misfolded proteins, and the dissociation of protein aggregates. Aside from their constitutive expression, these proteins can be significantly induced in response to various environmental, pathological, or physiological stimuli. HSPs are also involved in the induction of several pro-inflammatory cytokines, such as IL-2 and IL-15, and associated signaling pathways [12]. Heat-shock proteins (HSPs) play a crucial role in a variety of cellular maintenance processes, including the folding of newly synthesized polypeptides, assembly of protein complexes, degradation of misfolded proteins, and dissociation of protein aggregates. The role of HSPs in maintaining cellular integrity is crucial, as they are vital for helping cells manage different types of stress. When faced with challenges, like heat, hypoxia, oxidative stress, DNA damage, or the accumulation of misfolded proteins, the heat-shock response rapidly triggers the upregulation of multiple HSPs to stabilize the disrupted cellular environment, thus restoring cellular structure and metabolism. For example, in the plant kingdom, HSPs play a crucial role in protecting cells from various stresses, such as temperature extremes, heavy metals, and ozone stress [13,14].

Therefore, HSPs are generally activated by extreme temperatures [15], UV radiation, ozone, osmotic stress, and microgravity [16,17].

Gravity has played a crucial role in the development of terrestrial organisms. Thanks to experiments conducted in the microgravity environments of the US experimental space station, Skylab, the International Space Station, and the Tiangong space station (China), researchers have gained valuable insights into how organisms perceive gravity at the cellular, tissue, and organismic levels. These studies used a wide range of animal, cellular, and botanical models [18,19,20,21], revealing intriguing new insights into the role of HSPs in microgravity conditions. For instance, studies on cell cultures of Arabidopsis thaliana have shown that these cells can sense and react to spaceflight despite lacking the specialized cellular structures typically associated with gravity perception in whole plants. Specifically, researchers have observed that certain genes responsible for a subset of proteins and heat-shock factors (HSPs) are only activated in response to prolonged rotation and not during brief periods of microgravity experienced during spaceflight. This suggests that undifferentiated cells are capable of perceiving their gravitational environment. Furthermore, the activation of HSP genes in prolonged or simulated microgravity conditions supports the hypothesis that HSP-related proteins play a role in maintaining cytoskeletal architecture and signaling the shape of the cell [22].

In recent studies, carried out on board the ISS but using both Russian and American launch vehicles, the impact of extended space travel on human physiology was examined using Drosophila melanogaster as a model. These fruit flies, well known as a reliable model for spaceflight, have significantly contributed to the comprehension of various pathophysiological responses, including neurobehavioral, age-related, immune, cardiovascular, and multiomic changes in different tissues and developmental stages. Specifically, one study observed the upregulation of heat-shock protein genes (HSP70a, HSP70b, HSP68) in female flies exposed to simulated hypergravity, indicating the potential role of HSPs in maintaining proper protein folding and function under stressful conditions, thereby protecting the physiological systems of organisms from the detrimental effects of hypergravity [23,24].

Recent studies have also shown that under microgravity conditions, heat-shock proteins (HSPs) may trigger various signaling pathways related to stress response and cell survival, such as the nuclear factor kappa B (NF-κB), mitogen-activated protein kinase (MAPK), and tumor suppressor p53 pathways. The results suggest that changes in the expression of HSPs and NOX4 genes in response to microgravity conditions may signal cell pro-survival activation [25,26].

Despite the valuable findings from these studies, there is still a need to comprehensively understand the specific molecular pathways utilized by HSPs as part of the tissue stress response under microgravity conditions.

## 3. Cardiovascular System: The Role of HSPs in Endothelial Cell Survival

All living organisms on Earth have adapted to survive under the influence of gravity. Orbital spaceflight has shown that the absence of or a reduction in gravity profoundly affects eukaryotic organisms, including humans. Since endothelial cells are crucial in maintaining the functional integrity of the vascular wall and cardiovascular deconditioning has been described in astronauts, it is also interesting to assess whether microgravity affects endothelial function. Heat-shock proteins (HSPs) play a critical role in preserving cell viability and properly folding cytoskeletal proteins in response to microgravity conditions. Recent studies [27,28] have shown that in both human cutaneous microvascular endothelial cells (HDMECs) and human umbilical vein endothelial cells (HUVECs), placed in simulated microgravity using a rapid rotating wall vessel (RWV), HSP70 and HSP27 are essential for early adaptation to microgravity, as they protect the organism from cell death. Notably, in HUVECs, simulated microgravity triggers the sequential activation of various antioxidant proteins that ultimately counteract the increase in pro-oxidants, such as TXNIP. The regulation of HSPs in simulated microgravity appears to be linked to the stress pathway and cytoskeletal remodeling. This indicates how the cytoskeleton can translate the mechanical stress signal into a chemical signal, activating the stress pathway to preserve cell viability [29,30]. The increase in TXNIP could serve as a mechanism for containing cytoskeletal alterations, which would be vital for developing strategies against microgravity-induced muscle changes.

The possible practical application of the HSP response to microgravity conditions in future space missions could involve safeguarding the newly restructured cytoskeleton and improving endothelial survival under stress [31].

Further evidence from a study by Maier et al. [32] indicated that HUVECs and HMECs cultured for different time periods in rotary cell culture systems did not display apoptosis, which was linked to the rapid induction of the heat-shock protein HSP70. HSP70 has been shown to protect endothelial cells from apoptotic stimuli by acting downstream of cytochrome c release and upstream of caspase 3. Overall, microgravity appears to reversibly stimulate endothelial cell growth, as recent studies have indicated. This effect is associated with an overexpression of heat-shock protein 70 (HSP70) and a reduction in interleukin-1 alpha (IL-1alpha), a potent inhibitor of endothelial cell growth that promotes senescence [33,34]. Furthermore, endothelial cells not subjected to gravity loads undergo rapid cytoskeletal remodeling and, after a few days, significantly reduce actin through a transcriptional mechanism. It is hypothesized that the decrease in actin in response to microgravity represents an adaptive mechanism to prevent the accumulation of unnecessary actin fibers [35].

These studies suggest that simulated weightlessness is a stressor that increases the expression of HSP70, which could be beneficial for training astronauts by pre-adapting them to non-damaging stress exposures or other environmental factors to enhance the astronauts’ ability to tolerate weightlessness.

## 4. Muscular System: The Protective Role of HSPs Against Muscle Damage During Spaceflight

As for the muscular system, in microgravity, it undergoes adaptation processes due to the lack of need to support the full weight of the body. These processes lead to muscular remodeling, known as an atrophic response, i.e., a reduction in muscle mass, resulting in weakness and a total loss of motor function. The decreased muscle function increases the risk of the astronaut’s inability to perform physically demanding tasks while transitioning to a more demanding environment (e.g., Earth) [36,37]. The main changes in muscles exposed to microgravity include the following:

1. An overall reduction in muscle mass, leading to skeletal muscle atrophy and resulting in a decrease in functional performance. In this regard, several studies have shown a loss of volume of 7% in space missions lasting between 9 and 16 days [38];
2. A decline in protein synthesis and degradation of fine actin filaments due to microgravity and disuse, reducing contractile strength, speed, and muscle endurance. An increased loss of muscle strength has been observed, particularly in muscle groups involved in maintaining posture and walking (e.g., lower limb and trunk muscles) [39];
3. A qualitative and quantitative change in muscle fibers [40]. Researchers analyzed biopsies of the vastus lateralis muscle of eight crew members, obtained 3 to 16 weeks before launch and after landing (within three hours) from missions lasting between 5 and 11 days [41]. Comparing the samples obtained after landing and before the flight, the results showed a relative proportion between slow and fast fibers in favor of the latter and a reduced capillary density.

A study by Ishihara et al. examined the gene expression levels of heat-shock proteins (HSPs) in the slow twitch soleus and fast twitch plantar muscles of rats following hindlimb suspension or spaceflight. The goal was to understand how the gravitational stress from hindlimb suspension and actual spaceflight impacts the expression of these proteins in rats. The results revealed that hindlimb suspension and spaceflight both suppressed the mRNA expression levels of HSPs (specifically, HSP27, HSP70 and HSP84) in the slow-twitch soleus and fast-twitch plantar muscles of rats. This suggests that the mRNA expression levels of HSPs are influenced by mechanical and neural activity, and the decrease in HSP mRNA expression levels in slow twitch muscle following hindlimb suspension and spaceflight is linked to reduced mechanical and neural activity [42].

These findings have implications for future research and could enhance our comprehension of muscle atrophy and its prevention. Moreover, they may contribute to the development of methods or strategies to mitigate damage or muscle atrophy during prolonged spaceflight or bed rest, offering potential benefits for astronauts and individuals with physical limitations on Earth.

## 5. Bones: HSPs and the Challenging Adaptation of Bones to Microgravity

Over the millennia, the skeletal and muscular systems of humans have evolved to adapt to the environmental conditions and the force of gravity on Earth. The microgravity condition and the consequent reduction in the mechanical load on the bearing bones during spaceflight cause an increase in bone resorption and a decrease in bone synthesis, directly proportional to the duration of the space mission [43]. Physiologically, dynamic processes of resorption, renewal, and remodeling occur in bones. Such mechanisms are performed by different bone cytotypes, in turn regulated by several transcription factors, cytokines and growth factors (FGF, TGF-beta1), age, sex, physical activity, and drugs [44]. During long-term space missions, bone resorption increases by up to 50%, as does urinary calcium excretion, with an increased risk of kidney stone formation. Increased bone resorption also results in the suppression of PTH levels.

Furthermore, the effects of osteoporosis are similar to the changes occurring in the bones after long-term exposure to microgravity. There is an increase in osteoclastic activity and changes in trabecular plates that tend to perforate, thin, and lose their interconnection [45]. In simulated microgravity, there was a significant increase in the levels of heat-shock protein 60, heat-shock protein 70, superoxide dismutase 2, and cyclooxygenase 2. There was also a notable rise in RUNX family transcription factor 2 (RUNX2), which is crucial for the differentiation of osteoblasts, hypertrophy of cartilage at the growth plate, cell migration, and vascular invasion of bone, as well as osterix. This was observed in a population of human bone mesenchymal stem cells (bMSCs) when compared to normal gravity [46]. The gradual increase in different proteins is thought to be an adaptive response to compensate for the absence of mechanical load caused by weightlessness and to preserve cell viability. Additional research is required to fully comprehend the molecular mechanisms that drive cellular adaptation to these circumstances.

## 6. Liver: Hepatic Alterations Lead to an Upregulation of HSP70 Expression in Microgravity Conditions

The effects of microgravity on the liver include the induction of liver injury, inflammation, apoptosis, and oxidative stress. It also disrupts hepatic carbohydrate metabolism, leading to a diabetogenic phenotype, and affects hepatic lipid metabolism, leading to early non-alcoholic hepatic steatosis. These effects have been observed in experimental animals during actual spaceflight and in microgravity simulations on Earth [47]. Research indicates that spaceflight and simulated microgravity disrupt liver homeostasis in experimental animals, leading to liver injury, inflammation, apoptosis, and oxidative stress. Additionally, these conditions impair carbohydrate metabolism in the liver, potentially explaining the diabetogenic phenotype observed in astronauts. Spaceflight has also been shown to cause lipid accumulation in the liver of experimental animals, suggesting an increased risk of non-alcoholic hepatic steatosis among astronauts [48].

Recent studies have shown that spaceflight and simulated microgravity can significantly impact liver biotransformation capacity [49].

A study by Cui et al. investigated the effects of simulated weightlessness on mRNA and HSP70 protein expression in the liver of rats. The study was conducted on Wistar rats, using hindlimb-suspension tail-suspension models for different durations. The results suggest that weightlessness acts as a stressor for the liver, increasing HSP70 expression at both mRNA and protein levels. Simulated weightlessness, such as tail suspension, was found to significantly increase HSP70 mRNA expression levels in the liver of rats compared to controls. Specifically, the peak time of HSP70 expression appeared at 6 h of suspension, earlier than that of HSP70mRNA, followed by a steeper downward trend compared to HSP70mRNA [50].

These results suggest that the liver is susceptible to microgravity or simulated weightlessness stress, and that the regulation of HSP70 gene expression occurs not only at the transcriptional but also at the post-transcriptional level, including mRNA stabilisation and translation. During the early spaceflight phase, the expression of HSP70 mRNA and HSP70 in the liver increased, consistently with the characteristics of inducible HSP70. It is believed that the ability of cells to develop thermotolerance or stress tolerance, a state of transient and non-specific resistance to severe stress after mild heat shock or exposure, is mainly due to the presence of HSP70. This is hypothesised to result from stabilizing fundamental cellular processes, such as translation [51]. These findings may be helpful in training astronauts through pre-adaptation to non-harmful stress exposures or other environmental factors in order to foster their capacity to tolerate weightlessness.

## 7. Kidney: Exploring the Fine-Tuning of the Kidneys’ Adaptive Mechanisms to Microgravity and the Relationship Between HSP70 and Oxidative Stress

The way that body fluids are regulated is significantly affected under microgravity conditions and can be described as biphasic [52]. Initially, in the acute phase of the flight, the reduced gravitational force causes fluid displacement in the thorax and an increase in central blood volume, resulting in a characteristic ‘swollen face’ phenotype. Simultaneously, central venous pressure decreases due to reduced mediastinal pressure and increased thorax cavity compliance, along with the decrease in mechanical pressure on tissues and organs. During spaceflight, evaporation, oral hydration, and urinary excretion of fluids are generally reduced compared to the conditions on Earth. Despite this, cumulative water balance and total body water content remain stable if hydration, nutritional energy intake, and muscle mass protection are maintained. It is important to note that sodium management is significantly affected by microgravity [53]. A decrease in renal sodium excretion has been observed and is mainly attributed to increased sodium reabsorption due to the increase in filtration fraction accompanying the initial transient increase in glomerular filtration rate (GFR). Despite these adaptation mechanisms, there is no substantial microgravity-induced renal vascular dysfunction after spaceflight [54,55].

The altered fluid distribution in weightlessness leads to complex hemodynamic dysregulations, affecting total body water and sodium content. In a study of four astronauts, flight data were compared with data on the ground, and an increase in urodilatin, the main renal natriuretic peptide, was recorded, after a decrease initially observed during the first 24 h. After a saline infusion during the fifth day, when the new equilibrium in microgravity was well established, the renal response related to natriuresis and urodilatin levels in urine became attenuated compared to terrestrial data, although the reaction lasted longer in space [56]. This result probably indicates that the integrity of the regulatory mechanisms is maintained in microgravity and that other factors could influence the final result.

In the context of microgravity, it is crucial to consider that oxidative stress is promoted under such conditions. This phenomenon may arise from the body’s metabolism slowing down during spaceflight, acting as a ‘safety mode’ to adapt to low-energy conditions. As a result, there is a reduction in the production of pro-oxidant products, coupled with the loss of muscle and bone, leading to a negative energy balance. The challenging environment aboard the International Space Station impacts cell function by inducing oxidative stress, affecting multiple organs in space explorers. The kidneys, in particular, play a crucial role in the space environment, acting as regulators of oxidative stress while being highly susceptible to oxidative damage [57]. In a 2011 study, histopathological and molecular changes in rat kidneys were analyzed under weightlessness and resistance training conditions. The aim was to investigate the effects of long-term weightlessness on kidney tissue. The study found that a moderate HSP70 expression had a protective role under stress conditions. However, when HSP70 was overexpressed or released from the cell, it acted as a danger signal that could trigger cell necrosis. Notably, the HSP70 protein showed a significant increase, approximately 7-fold, in the rat kidney tissue compared to the control group, where minor or moderate HSP70 protein expression was observed. This suggests that the HSP70 protein could contribute to necrosis in kidney tissue in the absence of gravity [58].

Overall, prolonged exposure to increased oxidative stress during and after spaceflights, combined with radiation exposure, results in complex kidney damage. This affects both tubular and glomerular integrity, as well as the micro- and macrovascular function of the organ [59]. However, the long-term impact on kidney function after spaceflight remains uncertain. Further research is necessary to comprehend the effects of spaceflight on renal survival and potential glomerular dysfunction [60], particularly after long-term missions.

## 8. The Immune System: The Role of HSPs in the Adaptation of the Immune System to Microgravity

The space environment exposes astronauts to unique stressors during both short- and long-duration flights, leading to immune system dysregulation [61]. Research suggests that these stressors chronically increase the release of stress hormones, negatively impacting the adaptive immune system and potentially contributing to the reactivation of latent herpes virus in astronauts during and after spaceflight [62,63]. Elevated levels of stress hormones, such as cortisol and catecholamines, are linked to the activation of the hypothalamic–pituitary–adrenal axis and the sympathetic adrenal system, as well as changes in leukocyte subpopulations and function, including reduced T-cell and natural killer cell numbers and altered cytokine levels, all commonly observed during spaceflight [64]. A study by Pestov et al. analyzed the role of HSPs in the immune system in space. It explored how mechanical and environmental factors, such as launch vibrations and microgravity, affect the expression of HSPs in human lymphocytes. The findings reveal that mechanical environmental conditions can indeed influence the levels of HSP expression, including that of HSP70 and HSP27. For instance, the study demonstrates that cells express HSP27 at higher levels after being in microgravity for 24 h. Additionally, for HSP70a, the protein’s constitutive form, there was an increase in gene expression in cells that were subjected to a low-serum, low-temperature medium for 24 h prior to vibration [65].

These results suggest that challenging mechanical environments, such as those experienced during spaceflight, can impact cellular processes, including gene expression, in immune system cells. Furthermore, these findings indicate that HSPs play a role in the adaptive response of cells to specific stressful environments.

## 9. Conclusions

**Figure 1:**
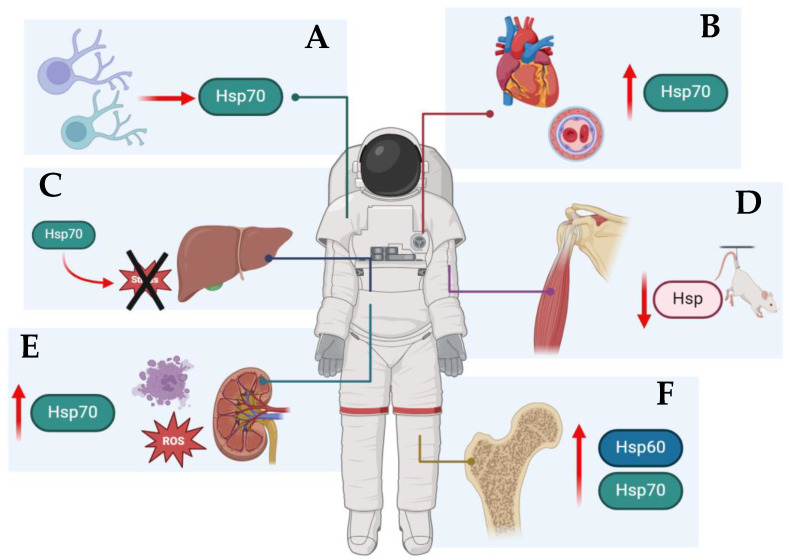
The figure schematizes the main roles that HSPs play in various organs and systems under microgravity conditions. (**A**) Immune system. The environmental factors in space affect the expression of HSPs in human lymphocytes. (**B**) Cardiovascular system. Microgravity stimulates endothelial cell growth by overexpressing heat-shock protein 70. (**C**) Liver. Simulated weightlessness increases Hsp70 expression. The presence of inducible Hsp70 in the liver increases during early spaceflight, providing cells with stress tolerance and stabilizing cellular processes. (**D**) Muscular system. HSP gene expression in rat muscles decreased during hindlimb suspension and spaceflight. (**E**) Kidneys. A moderate HSP70 expression is protective, but overexpression could trigger cell necrosis. (**F**) Bones. Simulated microgravity upregulated several vital proteins and transcription factors (such as RUNX2) in human bone mesenchymal stem cells compared to normal gravity.

Space medicine has experienced significant advancements over recent decades, thanks to the increasingly sophisticated technological capabilities for planning extended space missions and the recognition of the importance of applying the knowledge gained in space to benefit life on Earth. However, the attainment of increasingly ambitious goals requires a comprehensive exploration of the effects of stressful environments, such as space, on the human body. Over the years, numerous studies involving in vivo and in vitro models have revealed microgravity as a primary environmental factor in space, with both short-term and long-term effects on the human body. Based on established findings, this review aimed to investigate a generally underexplored aspect of space medicine: HSPs. While numerous studies on Earth have advanced our understanding of the role of HSPs, identifying the pathways in which they are involved in various processes and their key clinical implications, further research is necessary to investigate their role in the space environment, particularly under microgravity conditions. The literature review reveals that the role of HSPs in microgravity conditions has been primarily studied in connection to endothelial cells, the musculoskeletal system, liver, kidneys, and immune system [Figure 1; Table 1]. Across the analyzed studies, it is apparent that the upregulation of HSPs could be a valuable biomarker of the stress response and cellular adaptation during spaceflight.

These findings could serve as a valuable foundation for researchers looking to develop future studies related to space travel. These studies should aim to better understand the effects of microgravity on human physiology and create preventive measures to protect astronauts’ health. Furthermore, the findings could aid in identifying potential therapeutic targets for diseases on Earth that share similarities with the physiological changes observed during spaceflight. Therefore, this study could have practical implications for establishing strategies to maintain astronauts’ health and guarantee the success of future space missions. Additional research is necessary to comprehend the function of HSPs in various human body systems and organs exposed to microgravity, including the respiratory and nervous systems. Exploring these areas could lead to significant biomedical findings, especially anticipating long-duration missions and more challenging environments, such as the Moon and Mars.

## Figures and Tables

**Table 1:** HSPs and microgravity.

System/Organ	Proteins	Cells/Tissue	Action/Role or Event in Microgravity Condition	References
Cardiovascular system	TNXIP; HSP70; HSP27	Cytoskeleton; endothelial cells	The simulated microgravity increases TXNIP, which may contribute to cytoskeletal alterations, and HSP70 and HSP27 help protect endothelial cells from cell death	[26,27,28,29]
			Microgravity stimulates endothelial cell growth by overexpressing heat-shock protein 70 and reducing interleukin-1 alpha, which inhibits cell growth	[32,33]
			Endothelial cells not experiencing gravity undergo cytoskeletal changes, reducing actin to prevent unnecessary fiber accumulation	[34]
Muscular system	HSP27; HSP70; HSP84	Muscular tissue	HSP gene expression in rat muscles decreased during hindlimb suspension and spaceflight, indicating an influence of mechanical and neural activity on HSP mRNA levels	[41]
Bones	RUNX2	Bone mesenchymalstem cells	Simulated microgravity upregulated several key proteins and transcription factors (such as RUNX2) in human bone mesenchymal stem cells compared to normal gravity	[45]
Liver		Hepatocytes	Spaceflight can cause liver lipid accumulation and affect biotransformation capacity	[47,48]
	HSP70		Simulated weightlessness increases HSP70 expression in the liver of rats, particularly at 6 h of suspension	[49]
			The presence of inducible HSP70 in the liver increases during early spaceflight, providing cells with stress tolerance and stabilizing cellular processes	[50]
Kidneys			A moderate HSP70 expression has a protective role, but overexpression could trigger cell necrosis	[57]
	HSP70	Mitochondria; endothelial cells	The prolonged exposure to increased oxidative stress during and after spaceflights results in complex kidney damage	[58]
Immune system	HSP27; HSP70	Lymphocytes	Mechanical and environmental factors in space affect the expression of HSPs in human lymphocytes, e.g., microgravity and launch vibrations influence HSP expression levels	[64]

## Data Availability

Not applicable.
